# Repurposing approved drugs targeting *Leishmania infantum* 5-Methylthioadenosine Phosphorylase as anti-leishmanial candidates

**DOI:** 10.3389/fcimb.2026.1787791

**Published:** 2026-03-13

**Authors:** Yosser Zina Abdelkrim, Rafeh Oualha, Sonia Abbes, Isleme Khalfaoui, Thouraya Mejri, Mourad Barhoumi, Hela Abid, Emna Harigua-Souiai, Ikram Guizani

**Affiliations:** 1 Laboratory of Molecular Epidemiology and Experimental Pathology (LR11IPT04/LR16IPT04), Institut Pasteur de Tunis - University Tunis El Manar, Tunis, Tunisia; 2 Process Engineering Department, Institut Supérieur des Etudes Technologiques de Bizerte, Direction Générale des Etudes Technologiques, Tunis, Tunisia

**Keywords:** drug repurposing, *in vitro* validation, infectious diseases, Labetalol, *Leishmania* MTAP, structure-based drug discovery

## Abstract

**Introduction:**

Drug repurposing is a promising strategy for identifying new treatments against neglected tropical diseases such as leishmaniases, which are endemic in Asia, Africa, the Americas, and Southern Europe, offering the advantages of reduced development time and cost. In this context, computational and biochemical investigation of therapeutic targets plays a key role in guiding the selection of effective drug candidates.

**Methods:**

In this study, we investigated *Leishmania infantum* 5′-methylthioadenosine phosphorylase (LiMTAP) as a potential drug target by evaluating criteria defining such targets, including assayability, biochemical properties, and structural features enabling inhibitor selection. Trimeric 3D models of *Li*MTAP were generated, followed by virtual screening and docking of FDA-approved drugs. A robust miniaturized robotic assay was developed for recombinant *Li*MTAP to enable biochemical validation. Seven predicted drug candidates were subsequently tested in enzymatic and biological assays.

**Results:**

Two compounds—Labetalol and Halofuginone—inhibited *Li*MTAP activity with IC₅₀ values ranging from 200–400 µg/mL. The antileishmanial activity of all seven compounds was evaluated on extracellular promastigotes; four compounds (Dobutamine, Halofuginone, Labetalol, and Pentamidine) showed activity. Pentamidine and Dobutamine did not inhibit *Li*MTAP despite their anti-promastigote effects. Labetalol exhibited an IC_50_ of 29.67 µg/mL against extracellular promastigotes and showed no significant toxicity on THP-1 macrophages at effective doses (CC_50_ = 98.29 µg/mL). When tested on intracellular amastigotes, Labetalol demonstrated an IC_50_ value of 19.10 µg/mL.

**Discussion:**

This study confirms the *in silico* predictions through *in vitro* validation and highlights repurposed drugs as promising anti-*Leishmania candidates*.

## 1 Introduction

Leishmaniases are a group of vector-borne diseases transmitted by the bite of infected phlebotomine sand flies, posing a significant public health threat to over 1 billion people across more than 98 countries, with the most severe disease form responsible for an estimated 20,000 to 30,000 deaths annually. These diseases have been classified by the World Health Organization (WHO) as a neglected tropical disease (NTD) since 2011, reflecting the ongoing challenges in their control and management (WHO, 2025; Majoor et al., 2025).

Leishmaniases exhibit a wide spectrum of clinical manifestations, ranging from localized cutaneous lesions to severe visceral forms, which are fatal in approximately 95% of untreated cases. In the Indian subcontinent and East Africa, visceral leishmaniasis (VL) may also result in post-kala-azar dermal leishmaniasis (PKDL) following apparently successful treatment (Singh-Phulgenda et al., 2024). Existing therapies depend on invasive delivery of costly and toxic compounds, whose effectiveness is increasingly compromised by drug resistance (Majoor et al., 2025). Actually, no effective vaccine is yet available for human use (Gupta et al., 2025). Human prevention strategies rely solely on minimizing exposure to sand fly bites through repellents, insecticide-based interventions, or protective nets. Once infected, therapeutic intervention is the only option. Pentavalent antimonials remain the frontline therapy in many regions, although rising resistance poses a growing challenge. In high-income countries, liposomal amphotericin B is now the first-line treatment (Majoor et al., 2025). Despite their effectiveness, current therapies are hindered by several drawbacks, including invasive delivery methods, prolonged treatment courses, high financial burden, and increasing resistance (Ponte-Sucre et al., 2017).

Thus, the fight against NTDs, such as leishmaniases, is hampered by numerous challenges: limited therapeutic options, rising drug resistance, treatment-associated toxicity, and high costs that restrict their access in low-resource settings. These obstacles highlight an urgent need for innovative and more accessible treatment strategies.

A review of the limitations of current therapies and proposed strategies to address these issues, particularly through reducing toxicity, costs, and resistance, while exploring new administration routes and methods to shorten treatment durations, pointed to emergence of drug repurposing as a particularly attractive approach (Majoor et al., 2025). By redirecting already approved drugs towards new indications, this strategy drastically reduces development timelines and costs, while benefiting from existing safety and pharmacokinetic data. This reinforces the idea that repurposing can fast-track the availability of effective therapies in resource-limited environments. In this context, Miltefosine and Paromomycin are prominent examples of repositioned drugs successfully integrated into anti-leishmanial treatment regimens, and pentamidine and amphotericin B are well-established antiparasitic agents also effectively applied against *Leishmania* (Andrade-Neto et al., 2018). Particularly, pentamidine, which is a synthetic amidine derivative historically used as a first-line treatment for leishmaniases, is currently employed mainly as a second-line option due to toxicity and variable efficacy. It remains recommended for certain forms of cutaneous and mucocutaneous leishmaniasis, with cure rates and safety profiles differing across regions and *Leishmania* species (Zhang et al., 2025). It exhibits multifaceted and incompletely understood effects on *Leishmania* spp., including interactions with nucleic acids, tRNAs, kinetoplast DNA, mitochondrial function, and polyamine metabolism (Zhang et al., 2025).

In parallel, Majoor et al. (2025) underscored the relevance of combination therapies and nanocarrier-based delivery systems, such as liposomal amphotericin B, to enhance efficacy and mitigate toxicity. Yet, despite these advancements, the limited pipeline of novel anti-leishmanial drugs reflects the persistent gap in target discovery and validation (Majoor et al., 2025).

Leishmaniases remain neglected tropical diseases with limited therapeutic options. *In silico* approaches, including computational modeling and virtual screening, have emerged as promising tools to support drug repurposing efforts. A recent scoping review highlighted the potential of these strategies to accelerate the identification of effective compounds against *Leishmania* species (Scheiffer et al., 2024). The review systematically analyzed studies employing molecular docking, pharmacophore modeling, and other computational methods to identify candidate drugs for leishmaniases. It also emphasized the challenges and limitations of current *in silico* strategies, such as data availability and validation of predicted hits (Scheiffer et al., 2024).

Therefore, drug repurposing not only represents a cost-effective and time-efficient alternative to *de novo* drug development, but also constitutes a critical strategic pillar to address the unmet therapeutic needs associated with NTDs. Its integration with complementary approaches could further enrich the arsenal against leishmaniases and similar infections. These methodologies involve molecular docking, protein-inhibitor interaction analyses, virtual screening, QSAR models and most recently artificial intelligence (AI) models (Harigua-Souiai et al., 2018; Bhattacharjee et al., 2024; Oualha et al., 2024). In this context, we have previously employed structure-based strategies to virtually screen chemical libraries targeting the *Leishmania infantum* initiation factor 4A (LieIF), leading to the identification of compounds with *in vitro* selectivity indexes ranging from 19 to 38, underscoring their potential as novel anti-*Leishmania* agents (Harigua-Souiai et al., 2018). In contrast, our more recent work focused specifically on drug repurposing as we validated a machine learning pipeline, developed by our group, to identify FDA-approved drugs with predicted antileishmanial activity, and assessed the *in vitro* efficacy of ten of these candidates with promising results (Oualha et al., 2024). Similarly, Bhattacharjee et al. (2024) applied structure-based docking and profiling methods to reposition FDA-approved kinase inhibitors targeting *Leishmani*a MAP kinases, highlighting sorafenib and imatinib as potent candidates (Bhattacharjee et al., 2024).

The identification of novel parasite-specific molecular targets is a cornerstone of rational drug discovery for NTDs. It focuses on biochemical and metabolic pathways that show differences between pathogens and their host. Purine salvage, polyamine biosynthesis and thiol metabolism are key metabolic pathways currently explored as potential targets for cancer therapy, particularly because polyamine levels are elevated in proliferating cells (Gerner and Meyskens, 2004; Holbert et al., 2022). They are also targeted for drug development (Singh et al., 2012) and computer-aided drug-design approaches (Barazorda-Ccahuana et al., 2024) against diseases caused by *Trypanosomatidae* parasites.

Particularly, the 5’-Methylthioadenosine phosphorylase (MTAP) protein is among the enzymes involved in purine metabolism, playing a crucial role in purine and polyamine metabolism and in the methionine salvage pathway (Bacchi et al., 1991). The natural substrate of MTAP, the 5′-methylthioadenosine (MTA), which is generated during polyamine biosynthesis, is cleaved to adenine and 5′-methylthioribose-1-phosphate (Bacchi et al., 1991; Bertino et al., 2011). These products are then incorporated into the purine and methionine salvage pathways, respectively (Backlund and Smith, 1981). In addition, MTAP, the entry enzyme of the methionine salvage pathway, plays a key role in maintaining low intracellular levels of MTA, thereby supporting proper methionine recycling and preventing the accumulation of MTA, which can inhibit essential methylation processes (Backlund and Smith, 1981; Kirovski et al., 2011). Purine metabolism exhibits notable differences between parasites and their mammalian hosts. While mammals are capable of synthesizing purine nucleotides both through the *de novo* and salvage pathways, many parasites including Trypanosomatids rely solely on salvage mechanisms due to the absence of *de novo* biosynthetic routes (El Kouni, 2003). This metabolic reliance highlights the purine salvage pathway as a promising target for the development of selective antiparasitic therapies.

Several MTAP enzymes from archaeal, bacterial, and mammalian organisms have been investigated both in terms of enzymatic and structural characterization, as well as for inhibitor testing (Della Ragione et al., 1986, Della Ragione et al., 1990; Cacciapuoti et al., 2003; Singh et al., 2005; Guan et al., 2011a, Guan et al., 2011b). The research group of Schramm and colleagues characterized the bacterial MTAP-like enzyme 5′-methylthioadenosine/S-adenosylhomocysteine nucleosidase from *Escherichia coli* and **s**ynthesized small-molecule transition-state analogue inhibitors, e.g., Iminoribitol mimics of MTA. These compounds were evaluated as potent inhibitors, showing dissociation constants in picomolar to femtomolar ranges (Singh et al., 2005). Other studies further showed that synthetic analogues belonging to the Immucillins family — such as ImmA and ImmH — inhibited the recombinant nucleoside hydrolase NH36 from *L. infantum chagasi*, and reduced the multiplication of extracellular promastigotes and intracellular amastigotes *in vitro* without apparent toxicity (Freitas et al., 2015a, Freitas et al., 2015b). These findings support the idea that inhibitors developed against MTAP‑like enzymes may serve as promising candidates for antileishmanial chemotherapy.

Moreover, in *Trypanosoma brucei brucei*, a species closely related to *Leishmania*, MTAP has been identified as an important drug target, leading to the development of highly selective transition-state analogue inhibitors. Among these compounds, 5′-hydroxyethylthio-adenosine (HETA), an analogue of MTA, is highly metabolized by the Trypanosoma-MTAP in comparison to the mammalian counterpart (Bacchi et al., 1991; Sufrin et al., 1991). Growth inhibition assays revealed IC_50_ values of ≤1 μM for HETA, justifying its advancement to *in vivo* testing. In mouse infection models with *T. brucei brucei*, HETA produced cure rates between 70% and 90% (Bacchi et al., 1991). These findings highlight the relevance of MTA-related enzymes as chemotherapeutic targets in trypanosomatids. However, to our knowledge, *Leishmania* MTAP itself has never been investigated *in vitro* as a therapeutic target in *Leishmania*, underscoring the significance of characterizing MTAP as a potential druggable enzyme in *Leishmania* spp.

In this context, we previously performed a comparative analysis to investigate the structural and functional relatedness of *Li*MTAP protein to other known MTAPs. Sequence analysis revealed that *Li*MTAP shares a higher degree of identity with its homologs in *T. brucei* (*Tb*MTAP) and humans (*hu*MTAP) than with human purine nucleoside phosphorylase (*hu*PNP) (Abid et al., 2017). The *hu*MTAP structure was previously resolved at 1.7 Å (PDB ID: 1CG6), revealing a trimeric organization closely resembling that of mammalian purine nucleoside phosphorylase (*hu*PNP) (Appleby et al., 1999). Motif analysis using MEME further supported our finding, showing more conserved patterns and a closer similarity between the parasite proteins and *hu*MTAP than with *hu*PNP (Abid et al., 2017). Additionally, the three-dimensional structures of *Li*MTAP and *Tb*MTAP were predicted through homology modeling and compared to the crystal structure of *hu*MTAP (Abid et al., 2017), uncovering key structural divergence, particularly at the active site and C-terminal region, which suggested parasite-specific features exploitable for selective drug targeting (Abid et al., 2017). Taken together, the functional essentiality, host–parasite divergence, and accessibility of structural information make *Li*MTAP a promising target for antileishmanial drug development, particularly in the context of repurposing strategies.

In this study, we present a novel integrated approach that combines computational and biochemical analyses targeting *Li*MTAP protein with an *in vitro* biological validation to guide the selection of effective drug candidates against *Leishmania* parasites. We conducted a structure-based virtual screening and docking of FDA-approved drugs using trimeric models of *Li*MTAP and identified both known anti-leishmanial compounds and novel candidates, which were further investigated through biochemical and biological experiments for the selection of potential hit compounds. Thus, we developed a miniaturized assay for both biochemical characterization and screenings of the recombinant *Li*MTAP protein. Following this, active molecules were assessed for their cytotoxic effects on THP-1-derived macrophages, and tested on the extracellular and intracellular forms of *Leishmania* parasites. This integrated pipeline enables the identification of selective *Li*MTAP inhibitors, highlighting the FDA-approved drug Labetalol, and establishes a direct, previously unreported link between enzymatic inhibition of *Li*MTAP and cellular antileishmanial activity.

## 2 Materials and methods

### 2.1 *In silico* study

#### 2.1.1 Molecular modeling and docking simulations

The primary sequence of the *Li*MTAP protein was retrieved from the UniProt database (Consortium, 2021), using the accession number A4HSK5. The sequence was used as the target sequence to build 3D structure models of the monomeric *Li*MTAP using comparative modeling and AI-based approach. First, comparative modeling was performed using the Modeller software (Sali and Blundell, 1993) and the best five models were retained. In a second step, we built trimeric 3D structure models of the *Li*MTAP, as this protein is suspected to be active in its trimeric oligostate (Abid et al., 2017). We used the GalaxyHomomer web server (Baek et al., 2017) to derive a trimeric model from all monomers generated by the Modeller software. The best trimeric model was retained, and denoted MOD3. Then, the AlphaFold Protein Database (AFDB) was used to access the AI-inferred 3D structure of the target protein in its monomeric state (Varadi et al., 2022). Then, we used the AlphaFold2 software (Jumper et al., 2021) to generate the trimeric version of the *Li*MTAP model that we denoted AF3. Monomeric chains of the retained models AF3 and MOD3 of *Li*MTAP were validated through Ramachandran plots using the Swiss Model Server (Waterhouse et al., 2024), then targeted through molecular docking of the natural substrate. The crystal structure of the *hu*MTAP, co-crystallized with the natural substrate MTA and a co-factor molecule (SO_4_), was retrieved from the PDB (Berman et al., 2000), using the accession ID: 1CG6. It served as a reference structure for the optimization of the docking protocol.

First, the 1CG6 PDB file was cleaned through deletion of ligand and water molecules. Then, all three structure files (1CG6_cleaned, AF3 and MOD3), herein called receptors, were prepared for docking using AutoDock Tools suite of programs (ADT) (Morris et al., 2009). First, and in order to mimic the biological conditions of this reaction, we placed the SO4 cofactor molecules in the active site of LiMTAP structure models based on their spatial alignment with the 1CG6_cleaned structure. Then, hydrogen atoms were added and Gasteiger partial charges were calculated. All receptor files were saved in PDBQT format, required for docking with AutoDock vina (Trott and Olson, 2010). The MTA molecule was then prepared through hydrogen adding and gasteiger charges calculation, and saved in PDBQT format to serve as the ligand file. Docking of the ligand targeting the 1CG6 receptor was performed using AutoDock vina in multiple iterations, until the co-crystal pose of the MTA is reproduced. Docking parameters that led to the optimal redocking of MTA on 1CG6 were then used to perform the docking of the MTA and targeting AF3 and MOD3, and for the virtual screening.

#### 2.1.2 Virtual screening and molecules selection

We collated a chemical library composed of molecules presenting significant chemical similarity to the natural substrate. The MTA structure was submitted in a SMILES format to browse the DrugBank database (Wishart et al., 2018) for molecules presenting a Tanimoto similarity score higher than 0.2. A set of 487 molecules were selected. Then, literature review led to the identification of 32 additional compounds presenting a chemical similarity to the MTA and/or a validated activity against MTAP or methylthioadenosine nucleosidase (MTN) proteins from several organisms, but not against human MTAP. SMILES structure formats for molecules retrieved from publications (in PDF files) were generated using the OSRA server (Filippov and Nicklaus, 2009) based on the structure image in PNG/JPG format.

A total of 519 molecules collated in SMILES format composed the chemical library. For each molecule, the ligand preparation process was applied as previously described in the docking simulations section. For each ligand molecule, a PDBQT file was prepared. Docking was performed using the optimal parameters previously identified during the redocking of the MTA on the *hu*MTAP (1CG6). A search space of 28x28x28 Å was defined around the active site of the proteins, and the search algorithm leading to the best poses was the simulated annealing. A maximum number of 15 docking poses were obtained per molecule, reported after clustering of similar poses. Virtual screening of all 519 molecules was performed using a bash script for automation, targeting all three receptors: AF3, MOD3 and 1CG6.

The screened molecules were classified according to their docking scores. The top 20 molecules presented a range of docking scores significantly lower than the natural substrate, and thus were retained for further analysis. Among these 20 molecules, nine were identified as FDA-approved drugs for indications other than leishmaniases, and were thus selected for further analysis as part of a drug repurposing approach.

### 2.2 Biochemical validation

#### 2.2.1 Drugs and reagents

We purchased seven FDA approved drugs predicted *in silico* as potentially active against *Leishmania* parasites namely Leflunomide, Indapamide, Halofuginone, Labetalol, Flupiritine maleate, Pentamidine isethionate, and Dobutamine hydrochloride. The standard antileishmanial drug Amphotericin B was used as positive control. All compounds were commercially available in the MedChemExpress. Stock solutions of all compounds were prepared in dimethyl sulfoxide (DMSO; Sigma-Aldrich) as instructed by the manufacturer and kept in aliquots at -20 °C. Then, intermediate concentrations of the selected compounds were freshly prepared in water on the day of the experiment as needed. Detailed information including compound references, molecular weights and chemical structures are listed in the **Supplementary Material** (**Supplementary Table 1**).

#### 2.2.2 Expression and purification of recombinant *Li*MTAP

The MTAP gene was previously amplified by PCR from *L. infantum* genomic DNA, cloned into *TOPO- TA* Cloning plasmid, and verified by sequencing. The MTAP gene sequence was further subcloned into a pET-22b vector (Novagen, San Diego, CA, USA) cut with Xho*I* and *NdeI*. Expression and purification conditions were performed according to protocols previously optimized by our group, with minor modifications. Briefly, *Rosetta Escherichia coli* strain (Novagen), used in this study for the expression of His-tagged recombinant *Li*MTAP protein, were grown in LB medium (100 µg/mL ampicillin and 34 µg/mL chloramphenicol) at 37 °C and 180 rpm. Cultures (at OD600nm ~ 0.3–0.4) were induced with 0.3 mM IPTG for 2 h at 37 °C. Cells were collected by centrifugation (at 3500 rpm for 15 min at 4 °C) and stored at -20 °C until needed. Pellets were resuspended in a lysis buffer (300 mM NaCl, 10 mM imidazole, 20 mM Tris-HCl, pH 7.4) supplemented with protease inhibitor cocktail (Roche) and lysozyme (1 mg/mL), and incubated on ice for 30 min. Cells were disrupted by sonication (4 × 20 s) and centrifuged at 13,000 rpm for 30 min at 4 °C. The supernatant was loaded onto a 1 mL nickel-nitrilotriacetic acid (Ni-NTA) agarose column (Ni-NTA, Qiagen, Hilden, Germany) equilibrated with the lysis buffer. The column was washed twice, with a wash buffer 1 (300 mM NaCl, 20 mM imidazole, 20 mM Tris-HCl, pH 7.4), followed by a wash buffer 2 (Tris-HCl and 5% ASB) to reduce non-specific interactions and remove lipopolysaccharide (LPS). Proteins were eluted with elution buffer (300 mM imidazole, 10 mM Tris-HCl, pH 7.4). The purity of the proteins was checked on 12% SDS-PAGE gels that were stained with Coomassie blue. Purified proteins were stored at -80 °C after making 50% in glycerol (w/w). The proteins concentrations were determined using the Bio-Rad Protein Dye Assay (Biorad, Munich, Germany).

#### 2.2.3 *In vitro Li*MTAPase assays and statistical analysis

Recombinant *Li*MTAP protein containing His6 affinity tags, which was purified on nickel nitrilotriacetic-acid-agarose columns, was used for the establishment of a 96-Well plate miniaturized enzymatic assay.

We optimized the MTAPase assay based on our previously established tube-based protocol for *Leishmania* crude extracts, using conditions originally developed for extracts from the African trypanosome *Trypanosoma brucei* (Bacchi et al., 1991). We also relied on the published conditions used for the purified *E. coli* 5′-methylthioadenosine/S-adenosyl-homocysteine nucleosidase tested in the screening of synthetic Immucillins and DADMe-Immucillins inhibitor*s* (Singh et al., 2005). Optimization involved adjusting several reaction parameters, e.g., volume [50-100µl], temperature [25-37 °C], time [0-180min], and testing a range of MTA substrate concentrations [0-250µM]. Adenine formation was coupled to the equimolar conversion to 2, 8- dihydroxyadenine by xanthine oxidase. Thus, enzymatic activity was monitored at 305nm based on a reference curve generated from the adenine conversion using two commercial xanthine oxidase (XO) sources [MedChemExpress and Sigma] and various enzyme amounts [0.08-0.4 units per reaction].

Reaction mixtures contained 10ng/µl *Li*MTAP, 50mM potassium phosphate (pH 7.4), with the final reaction buffers including 200 µM MTA and 0.8 units of commercial XO (Sigma) per reaction, in a total volume of 100 µL per well. Reactions were incubated at 37 °C in 96-well microplates (Greiner, UV-STAR^®^ MICROPLATTE, 96 WELL, COC, F-BODEN BIO-ONE, KAMINFORM, TRANSP.), for up to 80 minutes. The reaction rates were determined by a linear regression fit of the initial linear phase of the curves, and measured in three independent experiments for each compound concentration. Data were analyzed with GraphPad Prism version 9.0.

A statistical analysis is essential to validate these MTAPase assay conditions for compound screening. In experiments testing the compounds, samples dissolved in DMSO were added to the reaction in different concentrations. Thus, DMSO levels [0-10%] were tested, and were further fixed at 5% (v/v) in screening reaction. Each plate included positive and negative controls in quadruplicate, allowing for statistical verification of results. The Z’ score (Zhang et al., 1999) was calculated for each plate to assess the quality and robustness of the assay, with values above 0.5 indicating reliable data (Harigua-Souiai et al., 2018). This metric ensures that the variability within control groups is minimal, supporting the validity of the experimental measurements.

#### 2.2.4 Biochemical screenings targeting *Li*MTAP recombinant protein

MTAPase biochemical screening assays were performed using 96-well microplates (Greiner, UV-STAR^®^ MICROPLATTE, 96 WELL, COC, F-BODEN BIO-ONE, KAMINFORM, TRANSP.). Columns 1 and 12 served as controls: wells A1–D1 and E12–H12 as negative controls (no enzyme), and E1–H1 and A12–D12 as positive controls (no inhibitor). Compounds were tested from columns 2 to 11 at a final concentration of 500 µM in duplicate on each enzyme *Li*MTAP and XO in presence of their natural substrates MTA and adenine respectively. The assays were independently repeated three times, and adenine release was measured for up to 80 minutes. Z’ scores were calculated for each plate; plates with Z’ < 0.5 were excluded. MTAPase inhibition percentage was determined using the corresponding equation (Harigua-Souiai et al., 2018), and compounds showing >15% inhibition without affecting XO activity (>5%) were selected for further analysis.

Selected hits were further tested with kinetic assays in the presence of 200 µM MTA, 5% DMSO, and increasing concentrations of compounds (0-1.25 mM). Results were reported as MTAPase reaction velocities. Therefore, the apparent MTAPase reaction velocity in the presence of increasing concentrations of the compounds were plotted as a nonlinear regression model of the logarithm of compound concentrations and the 50% inhibitory concentration (IC50) values were determined using GraphPad Prism version 9.0.1.

### 2.3 Biological validation

#### 2.3.1 Parasites

This study used cryopreserved laboratory stocks of *L. major* Empa-12 (MHOM/TN/2012/Empa-12), originally isolated from a zoonotic cutaneous leishmaniasis patient and previously described by Oualha et al (Oualha et al., 2019). The establishment and use of these strains were previously approved by the Ethical Committee of the Institut Pasteur de Tunis (approval reference: 2018/07/I/LR11IPT04). Briefly, in this study, thawed cryopreserved parasites were cultured at 22 °C in RPMI-1640/Glutamax medium (Gibco BRL, Germany) containing penicillin (100 U/mL) and streptomycin (100 μg/mL) supplemented with 10% heat-inactivated Fetal Bovine Serum (FBS) (Gibco BRL, Germany). Based on previously established growth kinetics, day 6 falls within the stationary phase of the culture (Oualha et al., 2024).

#### 2.3.2 *In vitro* antileishmanial assays

The selected compounds were assessed for their antileishmanial activity against promastigote and amastigote forms at varying concentrations. Negative controls consisted of untreated cells in 1% DMSO, while Amphotericin B served as a positive control.

First, the effect of these compounds on *Leishmania* promastigote viability was evaluated using the colorimetric 3-(4,5-dimethylthiazol-2-yl)-2,5-diphenyltetrazolium bromide (MTT) assay, which measures the reduction of MTT to formazan by mitochondrial succinate dehydrogenase in viable cells. Promastigotes (5 × 10^5^/well) in the stationary phase were seeded into 96-well plates and treated with two-fold serial dilutions of each compound (final 1% DMSO) (Abdelkrim et al., 2018; Harigua-Souiai et al., 2018; Oualha et al., 2024). The tested concentration ranges were: 1.56–200 µg/mL for Flupiritine, Indapamide, Leflunomide, Pentamidine, Dobutamine, and Labetalol; 1.03–66 µg/mL for Halofuginone. After 24 h at 22 °C, 25 µL of MTT (5 mg/mL) was added and incubated for 4 h. Formazan crystals were solubilized with 150 µL of DMSO, and absorbance was read at 570 nm. Because Dobutamine interfered with the MTT reagent, promastigote viability in its presence was assessed by manual cell counting using a Malassez hemocytometer. All treatments were performed in duplicate and repeated in three independent experiments. Promastigote viability (%) was calculated relative to controls.

The MTAPase-active compounds demonstrating anti-promastigote activity and showing no significant cytotoxicity against THP-1-derived macrophages (cytotoxicity detailed in the subsequent section) were further evaluated for their anti-amastigote activity as previously described (Harigua-Souiai et al., 2018; Oualha et al., 2024). Infected macrophages were treated with varying concentrations of the selected compound (e.g., Labetalol at 0, 6.25, 9.37, 18.75, 25, 37.5 and 50 µg/mL) for 24 h. After treatment, cells were fixed, stained with May-Grünwald-Giemsa, and examined by light microscopy (1000×). The percentage of infected macrophages and the mean number of intracellular amastigotes per 100 cells were determined. Amastigote survival was expressed relative to 1% DMSO controls.

Dose-response curves were generated, and promastigote and amastigote IC_50_ values were calculated by nonlinear regression using GraphPad Prism v9.0.1.

#### 2.3.3 Viability and cytotoxicity assessment on THP-1-derived macrophages

Human monocytic cell line THP-1, obtained from the American Type Culture Collection (ATCC, TIB-202) was used in this study. THP-1-derived macrophages were differentiated here as previously described (Abdelkrim et al., 2018; Harigua-Souiai et al., 2018; Oualha et al., 2024). Briefly, THP-1 cells were cultured in RPMI supplemented with 10% FBS, penicillin (100 U/mL), and streptomycin (100 µg/mL), and differentiated into macrophages with 25 ng/mL PMA for 24 h, followed by a 24 h rest. Cells were then exposed for 24 h to increasing concentrations of the tested compounds in the presence of 1% DMSO (final concentration). Their effects on cell viability and cytotoxicity were evaluated using both MTT (Harigua-Souiai et al., 2018; Oualha et al., 2024) and lactate dehydrogenase (LDH) assays (Aloui et al., 2016), as described below.

For the MTT assay, compounds demonstrating anti-promastigote activity were assessed for their effect on cell viability. Thus, cells were exposed to varying concentrations of Pentamidine (0.39–50 µg/mL), Dobutamine (3.12–400 µg/mL), Labetalol (1.56–200 µg/mL), and Halofuginone (2 ng/mL–66 µg/mL), with controls consisting of cells incubated in 1% DMSO-containing medium. Cell viability was assessed after 24 h by measuring absorbance at 570 nm and expressed as a percentage relative to untreated controls.

For the LDH assay, the cytotoxic effect of compounds exhibiting dual anti-*Li*MTAPase and anti-*Leishmania* activities with no significant impact in the MTT assay was further evaluated. This colorimetric test quantifies LDH enzyme released into the extracellular medium upon loss of membrane integrity, indicative of cell death. After 24 hours of incubation, culture supernatants were collected and incubated for 30 minutes at 37 °C with the reaction substrate provided in the Cytotoxicity Detection Kit LDH (Roche). Extracellular LDH activity was then quantified spectrophotometrically, and cytotoxicity was expressed as a percentage of cell death relative to untreated control cells.

Dose-response curves were plotted by mapping the logarithm of compound concentrations against the relative viability and cytotoxicity of THP-1-derived macrophages. CC_50_ values were calculated by nonlinear regression analysis using log-transformed concentrations versus percentage responses. Each experiment was performed in duplicate and repeated independently three times (GraphPad Prism version 9.0.1).

## 3 Results

### 3.1 Nine FDA-approved drugs were selected for anti-*Leishmania* screenings

We built the 3D structure models of the *Li*MTAP protein in its monomeric state using comparative modeling using Modeller. Best templates retained for this step were the PDB entries 4GLF, 1V4N, 2A8Y and 1WTA. All four templates presented resolutions lower than 2.45 Å and a similarity rate higher than 42% (**Supplementary Table 2**). The best-scoring model obtained with the 4GLF template exhibited the best DOPE score. Additionally, we retrieved the 3D model of the *Li*MTAP from the AlphaFold database. The structural alignment of both models was satisfactory, with minor divergence observed around variable loops and side chains packing (**Supplementary Figure 1**). In a second step, we generated the trimeric models of *Li*MTAP, herein called MOD3 and AF3, respectively. Ramachandran diagrams of the corresponding monomeric chains revealed satisfactory percentage of residues in favorable regions for each of the three models (**Supplementary Figure 2**). The MOD3 model presented 94.74% of residues in favorable regions, while the AF3 model achieved 96.71%. In order to compare both models, we performed a 3D alignment of AF3 and MOD3 using PyMOL, which yielded a significantly low RMSD of 0.72Å.

**Figure 1:**
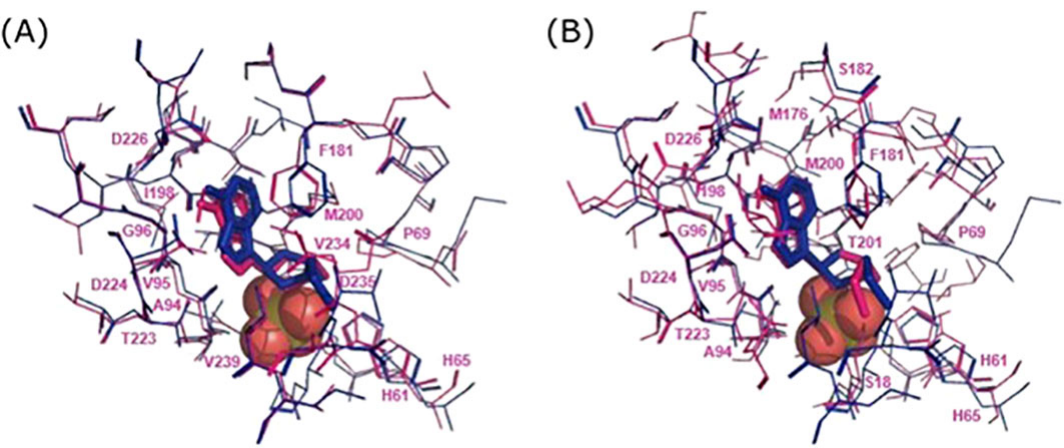
Docking poses of MTA on both models of *Li*MTAP in comparison with the crystal structure of the *hu*MTAP (PDB ID:1CG6). On both figures, structures of the MTA molecule co-crystallized with the huMTAP are shown in blue. Ligands are represented in licorice and interacting residues with the proteins are shown in lines. The SO_4_ co-factor is represented in orange spheres. **(A)** The docking pose of the MTA is represented in pink licorice on the Modeller (MOD3) structure model of *Li*MTAP represented by the interacting residues in pink lines with their corresponding labels. **(B)** The docking pose of the MTA is represented in pink licorice on the AlphaFold (AF3) structure model of *Li*MTAP represented by the interacting residues in pink lines with their corresponding labels.

Next, we performed a redocking of the MTA on the *hu*MTAP (1CG6), in the presence of the cofactor molecule (SO_4_). Iterative simulations with parameter tuning were performed. The retained set of docking parameters were those that yielded the reproduction of the co-crystal pose of the MTA, which validates our protocol (**Supplementary Figure 3**). These optimal parameters were retained for all subsequent docking simulations (**Supplementary Table 2**). Using these parameters, we docked the MTA on MOD3 (**Figure 1A**) and AF3 (**Figure 1B**) in the presence of the cofactor molecule. The docking poses with the closest conformation to the co-crystallized MTA with the *hu*MTAP (1CG6) were retained for further characterization (**Figure 1**).

**Figure 2:**
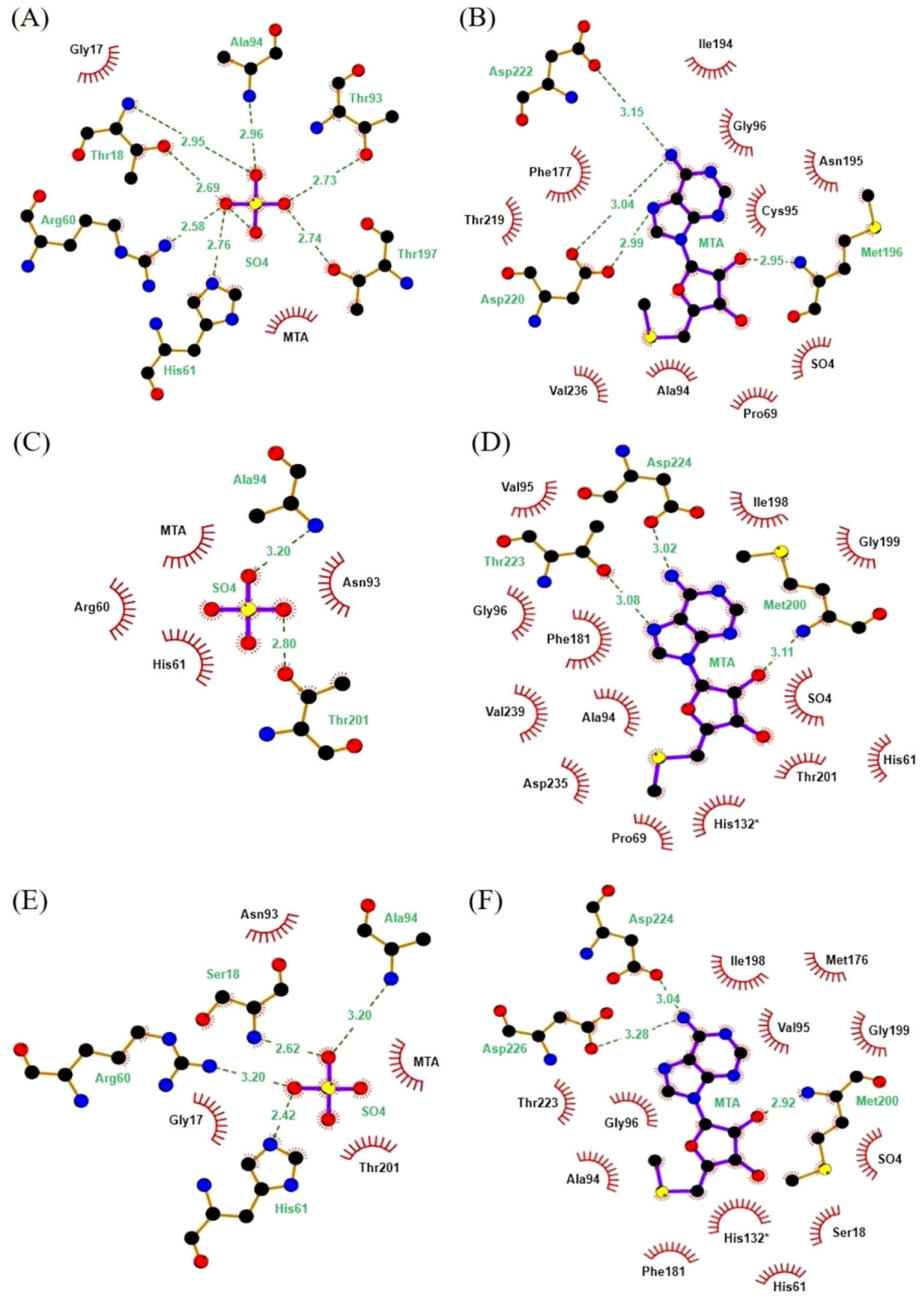
Interaction diagrams of the MTA and the SO4 molecules with the *hu*MTAP (1CG6) and the *Li*MTAP models (MOD3 and AF3). **(A, C, E)** correspond to the interaction diagrams of SO4 with 1CG6, MOD3 and AF3, respectively. **(B, D, F)** correspond to the interaction diagrams of MTA with 1CG6, MOD3 and AF3, respectively. Hydrogen bonds are shown in dashed green lines; residues and ligand atoms involved in hydrophobic interactions are represented with red arches; and residues marked with an asterisk (*) are those involved with the active site from chain C of the trimer.

Protein-ligand interactions between MTA and the *Li*MTAP (models MOD3 and AF3) in comparison with the *hu*MTAP (PDB ID: 1CG6) were analyzed using LigPlot+ (**Figure 2**). The interaction diagrams of SO_4_ with 1CG6, MOD3, and AF3 are shown in **Figures 2A, C, E**, respectively, while the interaction diagrams of MTA with 1CG6, MOD3, and AF3 are presented in **Figures 2B, D, F**, respectively. In *hu*MTAP, the cofactor was involved in six hydrogen bonds (H-bonds) with residues T18, R60, H61, T93, A94, and T197, along with a hydrophobic interaction with G17. The MOD3 model of *Li*MTAP presented conserved H-bonds interactions between the cofactor and residues A94 and T201, the structural equivalents of A94 and T197 in *hu*MTAP. However, the H-bonds involving residues R60, H61, and N93 (structural equivalents of R60, H61, and T93 in *hu*MTAP, respectively) were replaced by hydrophobic interactions. Additionally, interactions equivalent to those between SO_4_ and residues G17 and T18 in *hu*MTAP were absent. In contrast, the AF3 model performed better in reproducing the interactions between SO_4_ and the AS residues (**Supplementary Table 3**). Three out of six H-bonds observed in 1CG6 were conserved, specifically those involving R60, H61, and A94. The H-bonds between SO_4_ and residues T18, T93, and T197 of *hu*MTAP were replaced by hydrophobic interactions in AF3. Importantly, the hydrophobic interaction between the cofactor and G17 is preserved in AF3 (**Supplementary Table 3**).

The AF3 model, generated by AlphaFold, exhibited a higher conformational quality and reproduced key interactions with the natural substrate, reflecting the conservation of catalytic residues and essential substrate contacts required for MTA phosphorolysis, as described for the human ortholog (**Supplementary Table 3**). Noticeably, the H-bonds involving the MTA and residues D224 and D226, equivalent to D220 and D222 on the *hu*MTAP confirmed that the AF3 model presented the most optimal conformation of the AS residues, as compared to MOD3. Thus, the AF3 model was retained to be targeted throughout the virtual screening simulations.

The screening of the 519 molecules was performed using AutoDock Vina. Only 508 compounds were successfully docked, while 11 generated errors and were therefore eliminated. The selection process implemented in this study was straightforward. First, for each docked molecule, we selected the highest-ranked docking pose among those generated by AutoDock Vina. Then we selected the 20 best molecules exhibiting the lowest free energy of binding to the receptor. We obtained a set of scores ranging from -10.1 kcal/mol to -8.6 kcal/mol, which were significantly lower than the docking score obtained with the MTA molecule (-6.8 kcal/mol), with respect to the standard deviation of ±2.0 kcal/mol set as a threshold for significance by the authors of AutoDock vina (Trott and Olson, 2010).

Within the selected molecules, nine were FDA approved drugs. Out of these, only seven could be purchased for experimental validation, namely Leflunomide, Indapamide, Halofuginone, Labetalol, Flupiritine maleate, Pentamidine isethionate and, Dobutamine hydrochloride. The two remaining molecules were, Arbutamine and Debromohymenialdisine, and could not be tested in the present work.

### 3.2 The *Li*MTAP assay was suitable for biochemical screening *in vitro*

We expressed the protein in the *Rosetta Escherichia coli* strain and purified the soluble protein by nickel-nitrilotriacetic acid agarose chromatography. We estimated the protein to be greater than 90% pure after this column. In order to investigate its activity *in vitro*, we established a spectrophotometric assay in a 96-well plate format. A coupled enzymatic reaction was set up using two different commercial sources of XO, one from SIGMA and the other from MCE. Recombinant *Li*MTAP was assessed for its MTAPase activity in the presence of MTA as substrate and commercially available XO. The assay monitored adenine formation at 305 nm, which was further coupled to the equimolar conversion to 2,8-dihydroxyadenine by XO. We developed a robust and miniaturized assay suitable for kinetic analysis and *in vitro* biochemical screening, aiming to validate the activity of structure-based virtual screening hits. The results showed typical Michaelis–Menten kinetic profiles and revealed differences in the apparent kinetic values depending on the XO source (**Table 1**). Although the catalytic efficiency (kcat/KM) appeared higher with XO from MCE, the reactions performed with XO from SIGMA exhibited higher overall reaction velocities and improved data fitting (R² = 0.96 vs. 0.79). Therefore, the XO from SIGMA (**Supplementary Figure 4**) was selected for subsequent experiments to ensure that the coupling enzyme does not limit the rate of the MTAP-catalyzed reaction.

**Table 1:** Summary of the apparent kinetic values of *Li*MTAP recombinant protein.

Commercial XO	*K_M app_* µM	*V_max app_* µM.min^-1^	*K_cat app_* min^-1^	R^2^	Z score* 0%DMSO*	Z score* 5%DMSO*	Z score* 10%DMSO*
SIGMA	279.1±66.0	6.1±0.9	18.4±2.8	0.96	0.49	0.55	0.78
MCE	40.4±10,9	1,4±0.1	4.3±0.4	0.79	---	---	----

When XO from SIGMA was used, MTAP showed a higher apparent Vmax (6.1 µM·min⁻¹) and kcat (18.4 min⁻¹), with a lower apparent affinity (KM = 279.1 µM). In contrast, XO from MCE yielded lower apparent Vmax (1.4 µM·min⁻¹) and kcat (4.3 min⁻¹), but a higher affinity (KM = 40.4 µM). Although the catalytic efficiency (kcat/KM) was greater with MCE (0.106 vs. 0.066 min⁻¹·µM⁻¹), overall reaction rates and model fitting (R² = 0.79 vs. 0.96) were inferior. Therefore, XO from SIGMA was selected for the subsequent screening analyses to ensure that the coupling step does not limit the rate of the MTAP-catalyzed reaction.

*Results obtained with XO of SIGMA.

Final reaction mixtures were prepared in a total volume of 100 µl, containing 10 ng/µl *Li*MTAP, 50 mM potassium phosphate buffer (pH 7.4), varying MTA concentrations (0–250 µM), 5% DMSO, and 0.8 units of commercial XO (Sigma) per reaction. Reactions were incubated at 37 °C up to 80 minutes. These optimized conditions were further used to evaluate Z’-factor, a key statistical parameter to assess assay robustness in biochemical screening experiments conducted in 96-well plates. Columns 1 to 6 contained both full-signal controls (no inhibitor) and background controls (no enzyme). Wells [A1–H1], [A3–H3], and [A5–H5] served as negative controls with 0%, 5%, and 10% DMSO, respectively, while wells [A2–H2], [A4–H4], and [A6–H6] represented positive controls with 0%, 5%, and 10% DMSO, respectively. All experiments were performed in duplicate. Z’-factors were calculated for each plate based on the full-signal and background control groups. (**Table 1**). The calculated Z’-factors were 0.49, 0.55, and 0.78 at 0%, 5%, and 10% DMSO, respectively (**Table 1**). While the value at 0% DMSO was slightly below the conventional threshold (Z’ ≥ 0.5), both 5% and 10% DMSO yielded robust assays suitable for biochemical screening. Interestingly, assay quality improved with increasing DMSO, which may reflect enhanced substrate solubility or reduced signal variability. As compounds to be tested are dissolved in DMSO, it was essential to validate the assay under solvent-containing conditions. For subsequent biochemical screening, 5% DMSO was selected as the working condition, providing reliable assay performance while minimizing potential solvent-related effects.

### 3.3 Two out of seven drug candidates were selected *in vitro* as *Li*MTAP inhibitors using a 96-well plate biochemical screening assay

Compound screening was performed by monitoring *Li*MTAP activity in the presence of 500 μM of each compound, using 96-well plates in duplicate across three independent experiments. On the same plates, each compound was also tested in duplicate for its effect on XO activity using adenine as a substrate. A compound concentration of 500 μM was selected to maximize the detection of inhibitory or stimulatory effects, considering that the natural substrate MTA is present at ~200 μM for *Li*MTAP. To ensure that any observed inhibition was specific to *Li*MTAP and not due to interference with the coupling enzyme, each compound was also tested at the same concentration against XO activity using ~200 μM adenine as a substrate. This parallel control allowed us to distinguish specific *Li*MTAP inhibitors from non-specific enzyme inhibitors. Based on the biochemical screening, we calculated the percentage of inhibition for each compound and selected compounds inhibiting *Li*MTAP by more than 15% without affecting XO activity. Dobutamine showed a significant increase in OD305nm with xanthine oxidase, which prevented a clear interpretation of its effect on *Li*MTAP. Interestingly, we identified two—Labetalol and Halofuginone—that selectively inhibited *Li*MTAP activity. Both compounds were selected for further biochemical characterization.

### 3.4 Labetalol and Halofuginone inhibited *Li*MTPase activity *in vitro* in a dose-dependent manner

**Figure 3:**
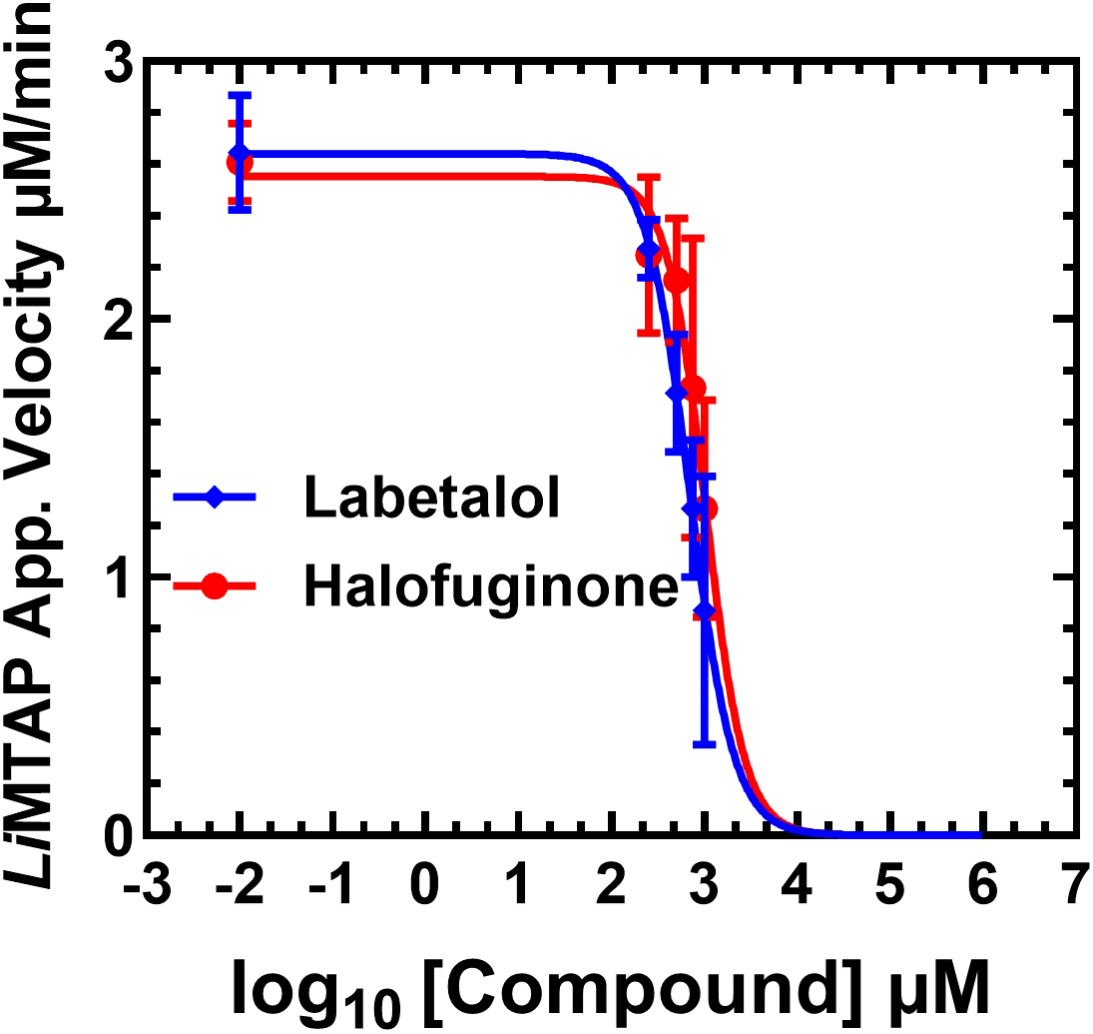
Reaction rates of *Li*MTAP protein in the presence of increasing concentrations of compounds. Points with error bars on each curve represent the mean ± standard deviation of apparent reaction velocities from three independent measurements performed using compound concentrations ranging from 0 to 1.25 mM. The data were fitted by plotting the logarithm of compound concentrations against the corresponding apparent reaction velocities and using a nonlinear regression model using GraphPad Prism version 9.0.1., with values estimated at a 95% confidence level.

In order to characterize the effect of the compounds on the MTAPase activity, we performed time courses for the MTAPase activity of *Li*MTAP at different concentrations of the compound in the 0 to 1.25 mM range, in the presence of 200 µM MTA and 5% DMSO. The amount of MTA hydrolyzed increased in a time-dependent manner and the corresponding MTAPase reaction rates for each compound concentration were determined and plotted (**Figure 3**). The apparent reaction velocity of the ATPase activity decreased in the presence of increasing amounts of the compound in a dose-dependent manner. The IC_50_ values were determined, and we obtained IC_50_ values of 697.3 µM (229.0 µg/mL) and 1021µM (406.8 µg/mL), respectively for Labetalol and Halofuginone.

Unlike the natural substrate MTA, which contains a rigid purine ring and a methylthio-ribose moiety essential for substrate recognition, Halofuginone (even with its rigid tricyclic structure) lacks key polar groups for mimicking these interactions. In contrast, labetalol, with its flexible structure and polar hydroxyl and amine groups, may better compete with MTA’s hydrogen bonding pattern, supporting its stronger inhibitory effect on *Li*MTAP protein.

### 3.5 Four candidate molecules exhibited anti-*Leishmania*l activity including two *Li*MTAP inhibitors

Although the target enzyme was derived from *L. infantum*, all *in vitro* antiparasitic assays were performed on *L. major*, a genetically related species. This choice was based on its well-established use as an *in vivo* laboratory model, and the practical availability of robust *in vitro* culture systems. Importantly, MTAP is highly conserved across *Leishmania* species, including *L. infantum* and *L. major*, supporting the relevance of cross-species testing. Thus, to confirm that *Li*MTAP inhibitors also had an effect on the parasite viability, we assessed the effect of all seven FDA-approved drugs, predicted *in silico* to have potential activity against *Li*MTAP, on the viability of *L. major* promastigotes at the stationary phase using an MTT assay after a 24h exposure. The standard anti-*Leishmania* drug, Amphotericin B, was used as a positive control. To this end, promastigotes in their stationary growth phase were seeded in 96-well plates (5 x 10^5^ parasites/well) and incubated for 24 h with increasing concentration of drugs as described in the Material and Methods section.

**Figure 4:**
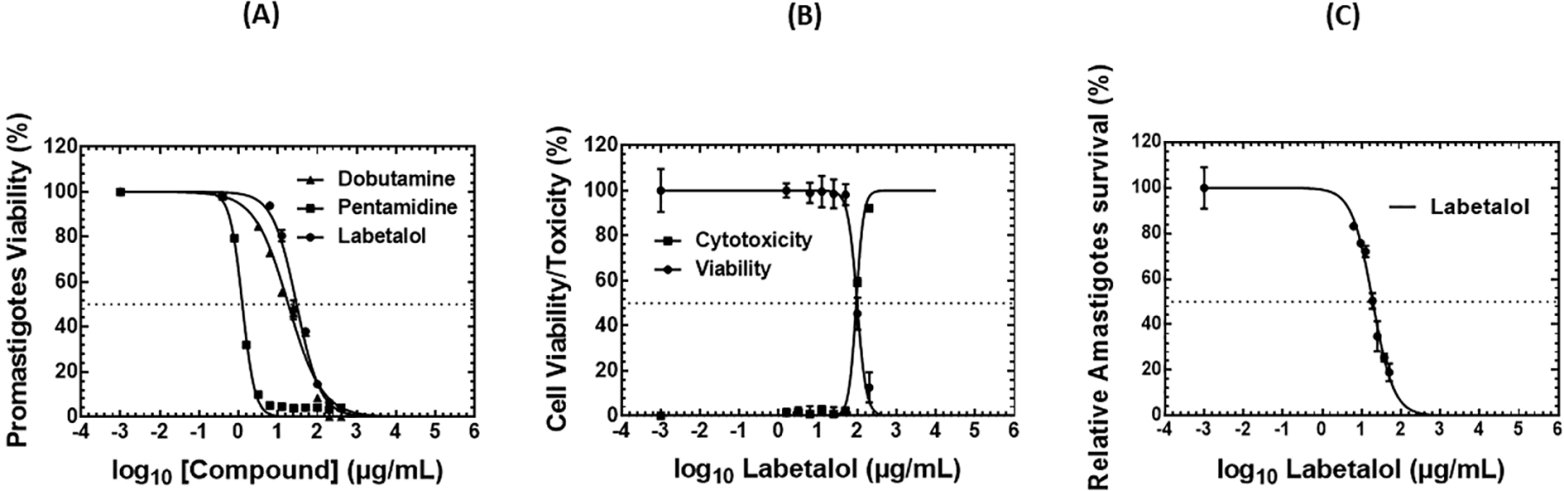
*In vitro* evaluation of the effect of the active FDA-approved drugs against extracellular and intracellular *L. major* parasites, and against THP-1-derived macrophages. **(A)***In vitro* evaluation of the effect of active FDA-approved drugs against *L. major* (Empa-12) promastigotes. Promastigotes in the stationary growth phase were seeded in 96-well plates at a cell density of 5x10^5^ parasites/well and incubated with increasing compound concentrations of 1.56–200 µg/mL for Flupiritine, Indapamide, Leflunomide, Pentamidine, Dobutamine, and Labetalol; and 1.03–66 µg/mL for Halofuginone. After 24h of incubation, parasite viability was evaluated with an MTT assay except for Dobutamine, which was assessed by manual cell counting using a Malassez hemocytometer due to its interference with the MTT reagent. Dobutamine, Pentamidine and Labetalol showed a dose response effect on promastigotes parasites viability. The results were expressed as the percentage of promastigote viability treated with compounds relative to parasites treated with 1% DMSO. Data are shown as the mean values ± SD of three independent biological replicates, each carried out in technical duplicates. **(B)***In vitro* viability and cytotoxicity effects of Labetalol, an *Li*MTAP inhibitor with anti-promastigote activity, against THP-1-derived macrophages. THP-1-derived macrophages were exposed to increasing concentrations of labetalol (1.56–200 µg/mL) for 24 (h) Cell viability was determined using the MTT assay, while cytotoxicity was assessed by measuring lactate dehydrogenase (LDH) release. Results are expressed as the percentage of viable cells relative to the 1% DMSO vehicle control, and LDH release as the percentage relative to the maximum LDH release (positive control). Data represent the mean ± SD of three independent experiments. **(C)***In vitro* activity of Labetalol against *Leishmania major* (Empa-12) intracellular amastigotes. THP-1-derived macrophages were infected with the (L) major Empa-12 strain for 24 hours, then incubated for an additional 24 hours with increasing concentrations of Labetalol (0, 6.25, 9.37, 18.75, 25, 37.5, and 50 µg/mL). After treatment, cells were fixed and stained using the RAL 555 rapid stain kit. The number of intracellular amastigotes per 100 infected macrophages was counted for each condition (1% DMSO or compound-treated). Parasite viability was expressed as the percentage of parasites in treated cells relative to the DMSO control. Data represent the mean ± standard deviation (SD) from three independent experiments.

Four compounds affected promastigote viability, namely Pentamidine, Dobutamine, Halofuginone and Labetalol. Pentamidine, Dobutamine, and Labetalol reduced the parasite viability with a dose-dependent manner (**Figure 4A**), in contrast Halofuginone showed an atypical dose–response profile (**Supplementary Figure 5**). Interestingly, Halofuginone and Labetalol, were previously identified in this study, as inhibitors of the MTAPase activity of *Li*MTAP. Dobutamine, which showed a positive effect on XO activity, was not evaluated for its potential inhibition of *Li*MTAP. IC_50_ values of 1.24 µg/mL, 18.24 µg/mL, and 29.67 µg/mL were obtained for compounds Pentamidine, Dobutamine and Labetalol, respectively. In the case of Halofuginone, an IC_50_ could not be calculated due to the compound’s atypical dose–response profile (**Supplementary Figure 5**). A clear reduction in viability was observed between 4.125 and 66 µg/mL, with values decreasing from approximately 50% to 5%. Below this range (0.002 to 4.125 µg/mL), a plateau phase was observed, with viability stabilizing at around 50%, suggesting a biphasic response. This biphasic profile may reflect two distinct modes of action: a cytotoxic effect at higher concentrations, and a cytostatic or metabolic modulatory effect at lower concentrations, limiting parasite proliferation without causing immediate cell death. The persistent reduction in viability at low doses could also result from sustained metabolic interference detectable by the MTT assay. Further complementary assays are necessary to clarify the compound’s mechanism of action. The three remaining compounds tested namely Leflunomide, Indapamide, and Flupiritine maleate did not exhibit significant inhibitory effects on *L. major* promastigote viability within the tested concentration range (0–200 µg/mL).

### 3.6 Labetalol, an *Li*MTAPase inhibitor, shows no cytotoxic effects on THP-1-derived macrophages at its IC_50_ concentration

To evaluate the safety profile of the active compounds, the effects of Labetalol, Pentamidine, Halofuginone, and Dobutamine on THP-1-derived macrophages were assessed in terms of viability or cytotoxicity.

First, their effects on the viability of THP-1 human macrophage-like cells were assessed by an MTT assay, except for Dobutamine due to reagent interference; however, microscopic observations revealed cell lysis and morphological alterations even at low concentrations, suggesting cytotoxicity of Dobutamine. Pentamidine showed notable decrease in macrophage viability at 25 and 50 µg/mL, with an estimated CC_50_ of 76.69 µg/mL indicating low cytotoxicity at concentrations effective against *L. major* promastigotes (**Supplementary Figure 6**). Halofuginone reduced cell viability across a range of 2 to 66 µg/mL; however, a plateau at lower concentrations precluded accurate CC_50_ determination, possibly due to compound instability or limited activity at low doses.

Labetalol affected cell viability only at the highest concentrations tested (100 and 200 µg/mL), with a calculated CC_50_ of 98.29 µg/mL. Importantly, no reduction in cell viability was observed at its IC_50_ concentration against promastigotes. Labetalol is first time described here as both a *Li*MTAP inhibitor and active against *Leishmania* promastigotes. As it did not affect THP-1-derived macrophages at its active concentrations on promastigotes, it was selected for further investigation. Thus, to further evaluate its potential cytolytic effect, an LDH (lactate dehydrogenase) release assay was performed. LDH is a stable cytoplasmic enzyme released upon cell membrane damage, making it a reliable marker of cell lysis and cytotoxicity. As shown in **Figures 4B**, Labetalol did not induce significant LDH release at concentrations up to 50 µg/mL, indicating preserved membrane integrity and low cytolytic potential at therapeutically relevant doses. A concentration-dependent increase in LDH release was observed only at higher doses, with a calculated CC_50_ of 93.4 µg/mL. Taken together these results confirm that Labetalol does not exert cytotoxic effects on THP-1-derived macrophages at its IC_50_ against *L. major* promastigotes.

### 3.7 Labetalol exhibits leishmanicidal activity against *L. major* intracellular amastigotes

Labetalol, which inhibits *Li*MTAPase and *Leishmania* promastigotes viability without affecting THP-1-derived macrophage toxicity, was evaluated for its efficacy against the intracellular amastigote form of *L. major*. Thus, THP-1-derived macrophages were infected with *L. major* Empa-12 (parasite-to-cell ratio 10:1) for 24 h and then treated with increasing concentrations of labetalol (6.25–50 µg/mL) for another 24 h. Cells were fixed, stained with RAL 555, and the number of intracellular amastigotes per 100 infected macrophages was quantified, with parasite viability expressed relative to 1% DMSO-treated controls. Labetalol induced a dose-dependent reduction in intracellular amastigote burden, with an IC_50_ value of 19.10 µg/mL (**Figure 4C**). These results suggest that Labetalol retains leishmanicidal activity against the intracellular form of the parasite, consistent with its observed efficacy against promastigotes and support its potential as a selective anti-leishmanial candidate targeting the MTAP protein.

## 4 Discussion

The present work extends our group’s ongoing efforts in computational and computer-aided drug discovery and repurposing against neglected tropical diseases. In contrast to our previous studies that relied either on chemical compounds’ screening through protein–ligand interaction modeling (Harigua-Souiai et al., 2018) or on drug repurposing conducted (independently of structural modeling) using ligand- and machine learning-based strategies (Harigua-Souiai et al., 2021; Oualha et al., 2024), the present work combines both approaches by integrating a structure-based modeling by addressing *Leishmania* Methylthioadenosine Phosphorylase (*Li*MTAP) as a potential therapeutic target (Abid et al., 2017), with a drug repurposing strategy supported by *in vitro* biochemical and biological validation. This integrated workflow strengthens the robustness of candidate prioritization by ensuring consistency between predicted binding behaviors and experimentally observed activities. Thus, we conducted structure-based virtual screenings and docking of FDA-approved drugs using trimeric models of *Li*MTAP and identified both known anti-leishmanial compounds and novel candidates, which are further investigated in this study *in vitro*. Then, we established a microplate-based biochemical test allowing the detection of two inhibitors of *Li*MTAP protein, namely Labetalol and Halofuginone, by testing seven clinically approved drugs (out of 9 predicted molecules in this study). The biological *in vitro* validation addresses all seven FDA approved drugs against the parasite in both extracellular and intracellular forms. Importantly, both active compounds on *Li*MTAP protein had an effect on *Leishmania* promastigotes, which provides a further confirmation of our strategy. Out of the five remaining molecules tested, two others exhibited a dose-response effect on *Leishmania* promastigotes, namely Dobutamine, and Pentamidine, with IC_50_ values of 18.24 and 1.24 µg/mL, respectively. Despite its established use as an anti-leishmanial drug species (Zhang et al., 2025), Pentamidine was included in our biochemical and cellular screening to evaluate its potential interactions with *Li*MTAP and to confirm its activity against *Leishmania* promastigotes in our experimental system.

Briefly, out of the four anti-promastigotes compounds identified here (Dobutamine, Halofuginone, Labetalol, and Pentamidine), both Dobutamine and Labetalol are described here for the first time (**Table 2**). Only Pentamidine and Labetalol could be further tested here on amastigote form of the parasite due to technical limitations. Particular attention was given to Labetalol in the subsequent analyses, as its anti-*Leishmania* activity is reported here for the first time (**Table 2**). Halofuginone induced a clear reduction in promastigote viability at concentrations ranging from 4.12 to 66 µg/mL. However, its atypical dose–response profile limited its suitability for downstream analyses. Despite its anti-promastigote effect observed for the first time in this study, Dobutamine was also not considered for further investigation due to its toxicity, including cell lysis and morphological alterations against THP-1-derived macrophages, even at low concentrations. Notably, Labetalol, was described herein for the first time as an MTAP inhibitor with both promastigote and amastigote anti-*Leishmania* effects *in vitro*, with no significant toxicity toward THP-1 macrophages (**Table 2**).

**Table 2:** Summary of the *in vitro* effects of the FDA-approved drugs: on the *in vitro* activity of *Li*MTAP, the viability of the promastigotes, THP-1-derived macrophages, and intramacrophage amastigotes.

FDA approved drugs	Leflunomide	Indapamide	Flupiritine maleate	Halofuginone	Pentamidine isethionate	Dobutamine hydrochloride	Labetalol
Anti-*Leishmania* data	Immune modulation *in vivo* (Solbach et al., 1995)	No anti-leishmanial data	No anti-leishmanial data	Theoretical and mechanistic arguments (Gill and Sharma, 2022)	Clinical applications (Zhang et al., 2025)	First time This study	First time This study
*Li*MTAPase IC_50_ µg/mL	NA	NA	NA	406.8 ± 22.40	NA	TND*	229.0 ± 0.67
Promastigotes IC_50_ µg/mL	NA	NA	NA	TND	1.24 ± 0.03	18.24	29.67 ± 1.50
THP-1-derived macrophages Cytotoxicity (LDH test) CC_50_ µg/mL	--	--	--	TND	--	<low concentrations	93.40 ± 1.46
THP-1-derived macrophages Viability (MTT test) CC_50_ µg/mL	--	--	--	TND	76.69 ± 17.86	TND	98.29 ± 2.95
Amastigotes IC_50_ µg/mL	--	--	--	--	--	--	19.10 ± 0.71

IC50 values, where applicable, of active drugs tested *in vitro* against *Li*MTAPase recombinant protein, promastigotes and intracellular amastigotes of *L. major*. CC50 values, where applicable, on uninfected THP-1-derived macrophages.

The mean of IC50 and CC50 values were determined from three independent experiments using Graphpad Prism, with values estimated at a 95% confidence level.

NA, Not Active.

TND, Tested but Not Determined.

--, Not Tested.

*, Interfere with the enzymatic test.

No anti-leishmanial data were reported, to our knowledge, for the three remaining non active compounds *in vitro*, namely, Leflunomide, Indapamide, Flupiritine maleate, except for Leflunomide (**Table 2**), for which a protective effect on the natural course of *Leishmania major*-induced disease in genetically susceptible BALB/c mice was described (Solbach et al., 1995). The study shows that leflunomide does not act as a direct antileishmanial compound, although it has been evaluated in the context of *Leishmania* infections. No significant *in vitro* leishmanicidal activity was reported which is consistent with our *in vitro* biological results. Its previously observed *in vivo* effects are therefore likely attributable to immunomodulatory activity rather than parasite-directed mechanisms (Solbach et al., 1995).

Taking into account our biochemical and biological findings, showing that Pentamidine and Dobutamine are not inhibiting the activity of *Li*MTAP despite their anti-promastigote effects, we hypothesize that these compounds do not target MTAP protein and may instead act on other pathways within the parasite, although no experimental *in cellulo* (cell-based assays) evidence is provided here to confirm this. In this context, Zhang and collaborators reviewed that Pentamidine is a multi-target drug whose leishmanicidal activity is primarily associated with mitochondrial dysfunction and kinetoplast DNA disruption, complemented by interference with polyamine transport and minor-groove DNA binding (Zhang et al., 2025). This aligns with our *in vitro* observations and further supports the conclusion that Pentamidine’s activity arises from mechanisms unrelated to MTAP inhibition. Actually, Pentamidine, which is an antiprotozoal and antifungal agent primarily used for the treatment of *Pneumocystis pneumonia* in HIV-infected patients (Wishart et al., 2018), exhibited activity against *Trypanosoma* and *Leishmania* species (Piccica et al., 2021), as well as certain fungal pathogens. Besides, Pentamidine isethionate has been administered intramuscularly for the treatment of cutaneous leishmaniasis caused by *L. guyanensis*, however, it has been reported to be associated with higher rates of treatment failure than when administered intravenously (Christen et al., 2018). Despite its therapeutic potential, the use of pentamidine is limited by its adverse effects, which include pancreatic toxicity leading to diabetes mellitus, central nervous system disturbances, and other systemic toxicities (Wishart et al., 2018). While our *in vitro* results confirm the potent anti-*Leishmania* activity of pentamidine with limited macrophage toxicity, its well-known systemic adverse effects continue to limit its clinical use. In the case of Dobutamine, it showed an inhibitory effect on both *Li*MTAP protein, and *Leishmania* promastigote viability, with cell lysis and morphological alterations even at low concentrations, suggesting its cytotoxicity. This drug is an β_1_-adrenergic agonist catecholamine used clinically to manage cardiac decompensation in patients with organic heart disease or following cardiac surgery (Wishart et al., 2018). Its potent cardiostimulatory action, though achieved with minimal vasoconstrictive or chronotropic effects, limits the range of concentrations that can be safely administered. Building upon these findings, our results should be considered further with caution, considering the dose-dependent activation of adrenergic receptors and potential host toxicity.

We also report here two novel inhibitors of *Li*MTAP *in vitro*, Labetalol and Halofuginone. We particularly reported the original anti-*Leishmania* activity of an additional FDA-approved drug, namely Labetalol. Labetalol exhibited inhibitory activity against both forms of *Leishmania* parasites, with low cytotoxicity toward THP-1-derived macrophages. This drug is an α- and β-adrenergic antagonist used in the treatment of hypertension, angina, and sympathetic overactivity syndrome in humans, and i**s** available in both injectable and oral tablet formulations. Labetalol i**s** a racemic mixture of two diastereoisomers, in which dilevalol, the R,R′ stereoisomer, constitutes approximately 25% of the mixture (Wishart et al., 2018). The anti-*Leishmania* activity of Labetalol is unlikely to be mediated through adrenergic pathways, which are absent in *Leishmania* species. The observed effect may instead involve alternative targets or nonspecific mechanisms affecting parasite viability, which requires further work to be determined. Interestingly, we report here that Labetalol inhibited the recombinant *Li*MTAP protein *in vitro*; Labetalol presents a flexible structure and contains polar hydroxyl and amine groups that may compete with MTA’s hydrogen bonding pattern, supporting its inhibitory effect on the MTAP protein. These results collectively support a mechanistic link between MTAP inhibition and Labetalol’s leishmanicidal activity, although additional target deconvolution studies are warranted. Notably, our group has also previously identified Acebutalol, a selective β1-receptor antagonist, as a novel anti-*Leishmania* agent (Oualha et al., 2024), further highlighting the potential of repurposing adrenergic modulators in leishmaniases treatment. This study also hypothesized that Labetalol could target Purine biosynthesis without excluding interactions with other targets. However, there is clear biochemical evidence for the interaction of the anti-MTAP-based inhibitors (virtually selected without prior reference to literature) with *Li*MTAP, and there is a correlation between the potencies of enzyme inhibition and leishmanicidal effects of the active molecules.

In contrast, Halofuginone inhibited the recombinant *Li*MTAP enzyme *in vitro* although with a high IC_50_ value (406.8 µg/mL). It also induced a clear reduction in promastigote viability but displayed an atypical dose–response behavior. To the best of our knowledge, there are theoretical and mechanistic arguments for Halofuginone as an antileishmanial agent (Gill and Sharma, 2022), but no published *in vitro* IC_50_ or *in vivo* efficacy specifically for *Leishmania* was reported. Halofuginone is a low molecular weight quinazolinone alkaloid and a potent inhibitor of collagen α1(I) and matrix metalloproteinase 2 (MMP-2) gene expression. Beyond its antifibrotic and antitumor properties, it has been developed for the treatment of scleroderma and received orphan drug designation from the U.S. FDA in 2000 (Wishart et al., 2018). It has been also shown to inhibit prolyl-tRNA synthetase activity by binding to glutamyl-prolyl-tRNA synthetase (EPRS), an effect reversible by exogenous proline (Keller et al., 2012). Considering its chemical structure and the requirement of phosphate as a cofactor in the MTAP-catalyzed reaction, it is possible that Halofuginone may interfere with phosphate binding rather than directly occupying the substrate-binding site of the enzyme. It is also possible that the compound may act differently *in cellulo* than *in vitro*, potentially interacting with other parasite targets that bind the compound with higher affinity than the native MTAP protein does. These alternative targets may not be essential for parasite survival, which could explain the limited or absent leishmanicidal effect observed for the tested concentrations.

These findings contribute to the growing body of research that applies computational drug repurposing strategies to neglected tropical diseases, consistent with the highlights of Scheiffer et al., which showed how *in silico* pipelines are cost-effective and constitute rapid tools to prioritize compounds with potential anti-leishmanial activity. They also emphasized that promising anti-leishmanial hits can emerge from compounds originally developed for unrelated therapeutic indications, illustrating that a wide range of pharmacological classes may hold potential for repurposing (Scheiffer et al., 2024). In this context, exploring methionine salvage pathway enzymes, such as MTAP, further broadens the druggable landscape beyond traditional targets. MTAP and related enzymes have been explored as therapeutic targets in trypanosomatids.

Previous work has focused on synthetic compounds and demonstrated that transition-state analogues, including HETA, exhibit potent activity against *Trypanosoma brucei brucei* MTAP and achieve high cure rates in murine models (Bacchi et al., 1991; Sufrin et al., 1991). Likewise, Schramm and colleagues showed that Immucillin derivatives inhibit MTAP-like nucleoside hydrolases such as NH36 in *L. infantum chagasi*, reducing parasite proliferation *in vitro* without significant toxicity (Freitas et al., 2015a, Freitas et al., 2015b). Although synthetic compounds generally require lengthy development pipelines before reaching the market as discussed in the review by Singh et al (Singh et al., 2023)—even in cases where they have previously undergone safety trials in humans (Freitas et al., 2015a, Freitas et al., 2015b)—the drug-repurposing approach remains considerably faster, as it relies on molecules that are already approved and therefore bypass many early regulatory steps (Singh et al., 2023). Our repurposing strategy specifically addresses the limited *in vitro* knowledge surrounding *Leishmania* MTAP protein, and partially bridged the gap, for the translation of computational hits into clinically viable candidates, through *in vitro* biochemical and biological validation of FDA approved molecules. Nevertheless, still, additional studies—including structure–activity relationship optimization, pharmacokinetic profiling, and *in vivo* efficacy testing—are essential before MTAP-targeting compounds can progress toward therapeutic development. On the other hand, this points out that future research should also prioritize multi-target drug repurposing strategies to overcome resistance and improve therapeutic outcomes. MTAP inhibitors may also be explored in combination with approaches targeting other validated *Leishmania* pathways to achieve synergistic effects, increase treatment efficacy and tolerance, reduce treatment duration and cost, limit the emergence of drug resistance, and broaden the anti-leishmanial arsenal.

To conclude, our strategy provides a dual layer of validation, linking parasite viability to direct enzymatic inhibition, thereby reinforcing the rationale for targeting *Li*MTAP in drug repurposing pipelines. Moreover, by coupling enzyme-specific assays with the phenotypic evaluation on promastigote and amastigote forms, this study bridges molecular mechanisms with therapeutic perspectives and highlights the translational potential of FDA-approved drugs for leishmaniasis therapy. Overall, these results underscore the value of integrating computational prioritization with biochemical and cellular validation to accelerate the identification of promising anti-leishmanial candidates. Together, these studies illustrate the complementarity of different computational strategies and highlight their potential in accelerating the discovery of effective anti-leishmanial agents.

## Data Availability

The original contributions presented in the study are included in the article/**Supplementary Material**. Further inquiries can be directed to the corresponding authors.
