# Supplementary material for: Repurposing approved drugs targeting Leishmania infantum 5-Methylthioadenosine Phosphorylase as anti-leishmanial candidates
Source: Front Cell Infect Microbiol. 2026 Mar 13;16:1787791. doi: 10.3389/fcimb.2026.1787791 (PMC13021629; doi:10.3389/fcimb.2026.1787791)
Supplement: Supplementary file 1 [file DataSheet1.pdf]

Repurposing approved drugs targeting *Leishmania* 5' MethylThioAdenosine Phosphorylase (*Li*MTAP) as anti-leishmanial candidates

Yosser Zina Abdelkrim[(1,2)*], Rafeh Oualha[(1)], Sonia Abbes[(1)], Isleme Khalfaoui[(1)], Thouraya Mejri[(1)], Mourad Barhoumi[(1)], Hela Abid[(1)], Emna Harigua-Souiai[(1)], Ikram Guizani[(1)]*

[1] Laboratory of Molecular Epidemiology and Experimental Pathology (LR11IPT04/LR16IPT04), Institut Pasteur de Tunis - University Tunis El Manar, Tunis 1002, Tunisia

2 Process Engineering Department, Institut Supérieur des Etudes Technologiques de Bizerte, Direction Générale des Etudes Technologiques, Tunis, Tunisia

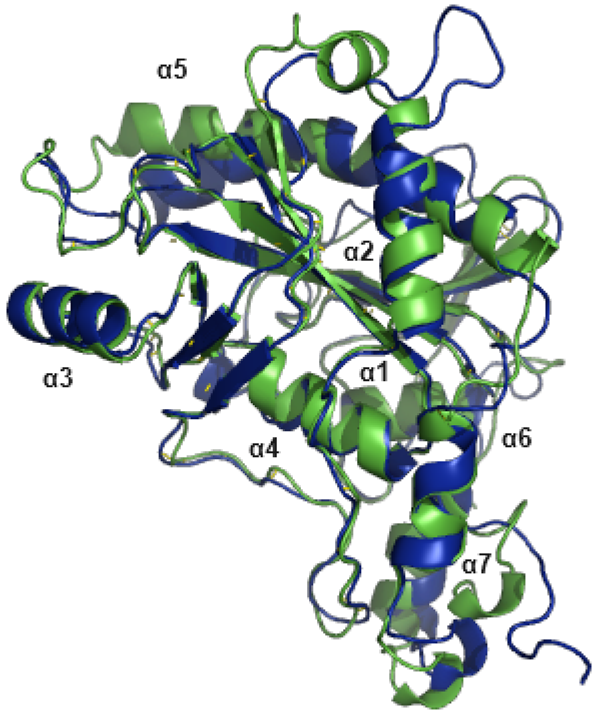

Supplementary Figure 1. Alignment of the monomer models of *Li*MTAP. The Modeller model (MOD) is shown in blue and the AlphaFold model (AF) is shown in green.

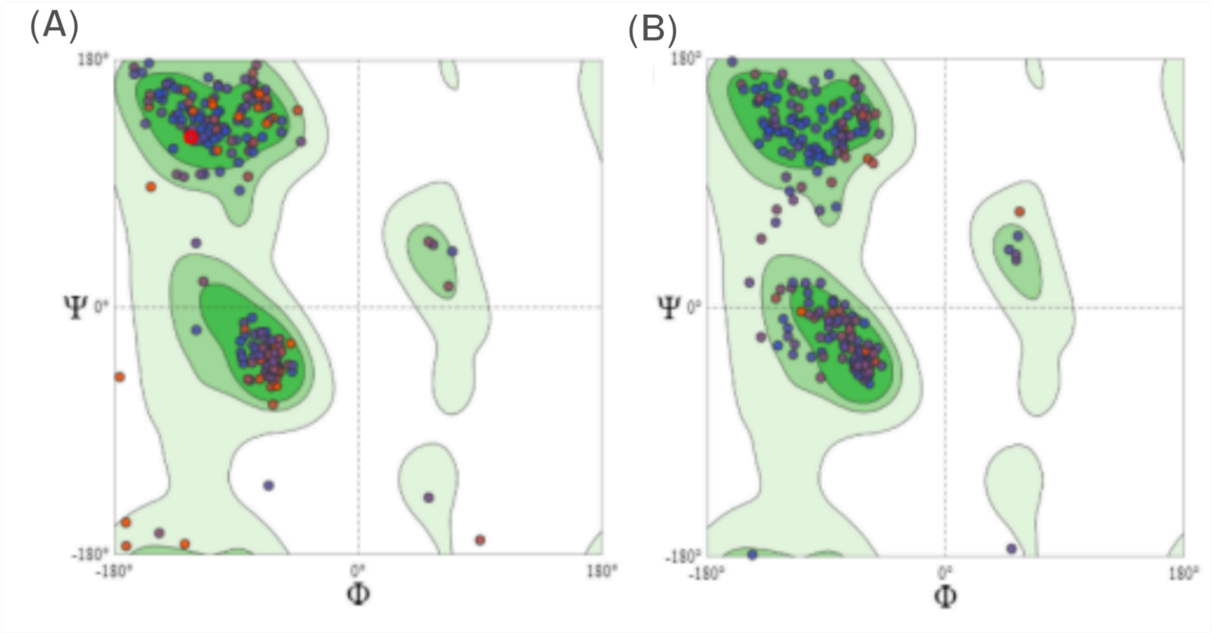

Supplementary Figure 2. Ramachandran diagrams of LiMTAP models obtained using the Swiss Model Server. (A) Diagram of the MOD3 model; (B) Diagram of the AF3 model.

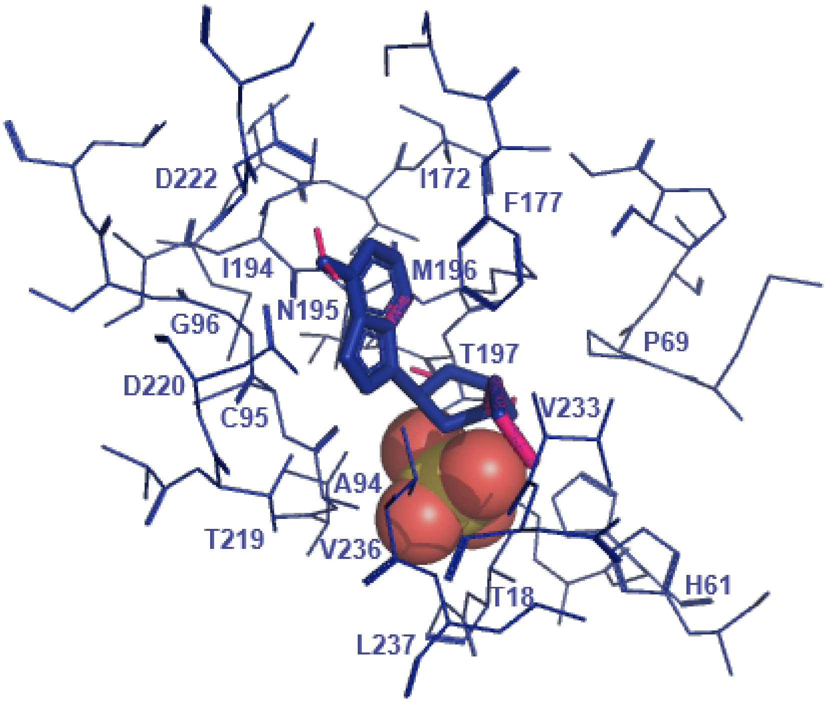

Supplementary Figure3. Alignment of the re-docked MTA on the huMTAP (PDB: 1CG6), with the co-crystallized molecule. The co-crystal MTA is shown in blue and the docking pose is shown in pink.

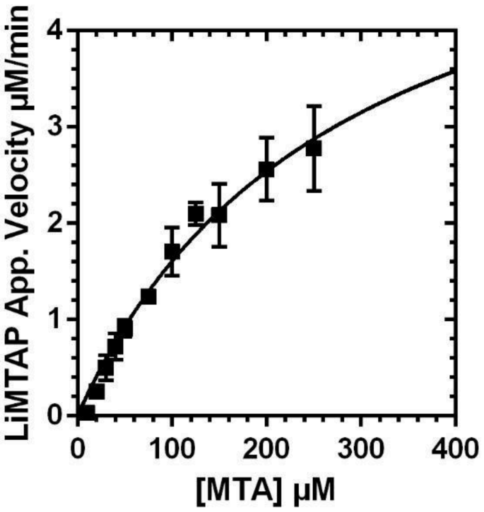

Supplementary Figure 4. Apparent MATPase reaction velocities of *Li*MTAP with different concentrations of the substrate MTA using commercial Xanthine oxidase from Sigma. The means and standard deviations are shown for at least three independent experiments. The best-fit values for Vmax and Km are reported in the manuscript and were

estimated using nonlinear regression, with a 95% confidence level. All the reactions were performed in the presence of 5% DMSO and 330 nM *Li*MTAP and 0.8 units of commercial XO (Sigma).

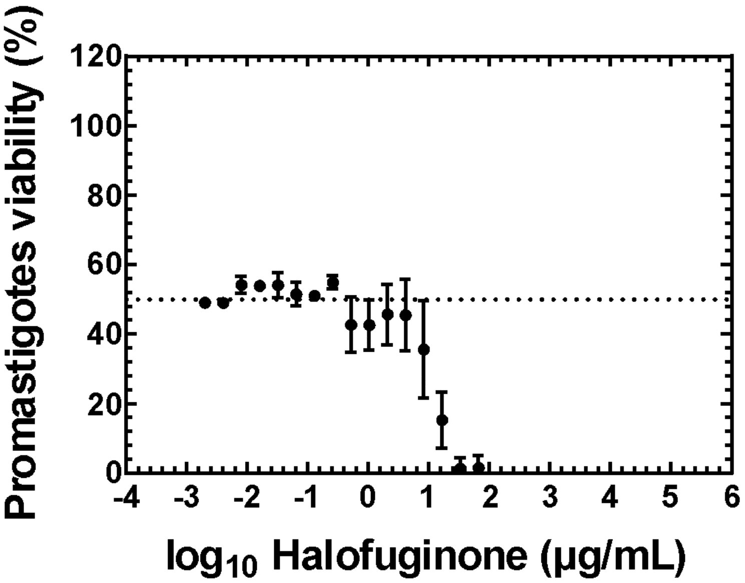

Supplementary Figure 5. *In vitro* evaluation of the effect of Halofuginone against *L. major* (Empa-12) promastigote. Promastigotes in the stationary growth phase were seeded in 96-well plates at a cell density of $5x10^5$ parasites/well and incubated with increasing concentrations of Halofuginone (1.03–66 µg/mL). After 24h of incubation, parasite viability was evaluated with an MTT assay. The results were expressed as the percentage of promastigote viability treated with compounds relative to parasites treated with 1% DMSO. The IC_{50} could not be calculated due to the compound's atypical dose–response profile. Data are shown as the mean values ± SD of three independent experiments carried out in technical duplicates.

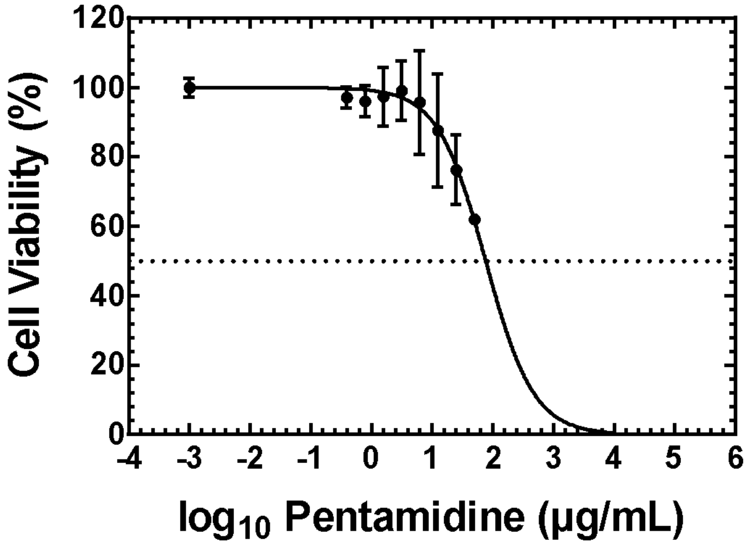

Supplementary Figure 6. Assessment of *in vitro* viability of Pentamidine on THP-1-derived macrophages. THP-1-derived macrophages were exposed to increasing concentrations of Pentamidine (0.39–50 µg/mL), for 24 h. Cell viability was determined using the MTT assay. Results are expressed as the percentage of viable cells relative to the 1% DMSO vehicle control. Data represent the mean ± SD of three independent experiments.

Supplementary Table 1. List, references, molecular weights and structures of FDA-approved Drugs used in the *in vitro* tests

Drug Name	Reference	Molecular weight (g/mol)	Structure
Leflunomide	HY-B0083	270.21	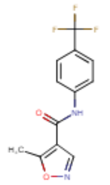
Indapamide	HY-B0259	365.8	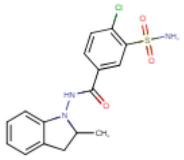
Halofuginone	HY-N1584	414.7	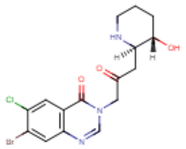
Labetalol	HY-121383	328.4	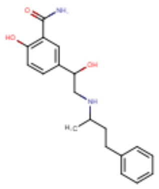
Flupiritine maleate	HY-17001	420.4	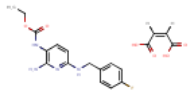
Pentamidine isethionate	HY-B0537B	592.7	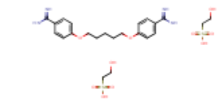
Dobutamine hydrochloride	HY-15746	337.8	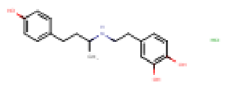

(*) All compounds were purchased from MedChemExpress

frontiers

Supplementary Table 2. Docking parameters retained for docking simulations using AutoDock Vina

**Parameters**	**Value**
AS center X	2.969
AS center Y	16.799
AS center Z	11.116
AS size X	31
AS size Y	29
AS size Z	30
Energy range	3
Exhaustiveness	16
Max number of binding modes	10
Seed	40

Supplementary Table 3. Active site residues of *Li*MTAP involved in interactions with the MTA substrate

1CG6	—	—	P69[b]	A94[b]	C95*	G96[a]	—	F177*	I194[a]	N195[a]	M196[b]	—	T219[a]	D220[a]	D222[a]	—	V236[b]
MOD3	—	H61[b]	P69[b]	A94*	V95[a]	G96[a]	—	F181*	I198[a]	G199[a]	M200[b]	T201[b]	T223[a]	D224[a]	—	D235[b]	V239[a]
AF3	S18[b]	H61[b]	—	A94*	V95[a]	G96[a]	M176[a]	F181[b]	I198[a]	G199*	M200[b]	—	T223[a]	D224[a]	D226[a]	—	—

Residues shown in red are involved in hydrogen bonds (H), whereas those in black are involved in hydrophobic interactions. Residues labeled (a) interact with the adenine base of MTA, while those labeled (b) interact with the ribose moiety of MTA. Residues marked with an asterisk (*) interact simultaneously with both the adenine base and the ribose of MTA.
